# Ticagrelor Improves Endothelial Function by Decreasing Circulating Epidermal Growth Factor (EGF)

**DOI:** 10.3389/fphys.2018.00337

**Published:** 2018-04-06

**Authors:** Francesco Vieceli Dalla Sega, Francesca Fortini, Giorgio Aquila, Rita Pavasini, Simone Biscaglia, Davide Bernucci, Annamaria Del Franco, Elisabetta Tonet, Paola Rizzo, Roberto Ferrari, Gianluca Campo

**Affiliations:** ^1^Department of Medical Sciences, University of Ferrara, Ferrara, Italy; ^2^Maria Cecilia Hospital, GVM Care & Research, E.S. Health Science Foundation, Cotignola, Italy; ^3^Department of Morphology, Surgery and Experimental Medicine, University of Ferrara, Ferrara, Italy; ^4^Cardiovascular Institute, Azienda Ospedaliero-Universitaria di Ferrara, Cona, Italy; ^5^Laboratory for Technologies of Advanced Therapies, University of Ferrara, Ferrara, Italy

**Keywords:** epidermal growth factor (EGF), eNOS, ticagrelor, endothelial function, stable coronary artery disease (SCAD), chronic obstructive pulmonary disease (COPD), EGF

## Abstract

Ticagrelor is one of the most powerful P2Y_12_ inhibitor. We have recently reported that, in patients with concomitant Stable Coronary Artery Disease (SCAD) and Chronic Obstructive Pulmonary Disease (COPD) undergoing percutaneous coronary intervention (PCI), treatment with ticagrelor, as compared to clopidogrel, is associated with an improvement of the endothelial function (Clinical Trial NCT02519608). In the present study, we showed that, in the same population, after 1 month treatment with ticagrelor, but not with clopidogrel, there is a decrease of the circulating levels of epidermal growth factor (EGF) and that these changes in circulating levels of EGF correlate with on-treatment platelet reactivity. Furthermore, in human umbilical vein endothelial cells (HUVEC) incubated with sera of the patients treated with ticagrelor, but not with clopidogrel there is an increase of p-eNOS levels. Finally, analyzing the changes in EGF and p-eNOS levels after treatment, we observed an inverse correlation between p-eNOS and EGF changes only in the ticagrelor group. Causality between EGF and eNOS activation was assessed *in vitro* in HUVEC where we showed that EGF decreases eNOS activity in a dose dependent manner. Taken together our data indicate that ticagrelor improves endothelial function by lowering circulating EGF that results in the activation of eNOS in the vascular endothelium.

## Introduction

Ticagrelor is one of the most powerful P2Y_12_ inhibitor (Motovska et al., 2018). Unlike other P2Y_12_ inhibitors, ticagrelor exerts some pleiotropic effects acting mainly on endothelial function in several settings (Schnorbus et al., 2014; Li et al., 2016; Campo et al., 2017; Kim et al., 2017). In a recent randomized clinical trial, in patients with concomitant stable coronary artery disease (SCAD) and chronic obstructive pulmonary disease (COPD) undergoing percutaneous coronary intervention (PCI), we demonstrated that 1 month treatment with ticagrelor, as compared to clopidogrel, is associated with an improvement in markers of endothelial function, such as a reduction of endothelial apoptosis and endothelial nitric oxide (NO) production, a reduction in ROS levels in PBMC as well as a more effective reduction of platelet reactivity (Campo et al., 2017).

At the state of the art the reason of the effect of ticagrelor on endothelial function is not completely characterized. One of the possible mechanism behind its pleiotropic effects could be related to the ability of ticagrelor to increase adenosine plasma concentration by inhibiting the adenosine type-1 equilibrative nucleoside transporter (ENT1) (Sumaya and Storey, 2017). Nonetheless, this view has been argued by some studies showing that ticagrelor is not always able to increase adenosine plasma concentration (van den Berg et al., 2015). This implies that increased plasma adenosine, at least in some circumstances, may not represent the only mechanism by which ticagrelor improves endothelial function and that other molecular targets may be involved.

It is well-established that circulating cytokines, such as TNF-α, cause increased endothelial apoptosis and inhibit eNOS activity in endothelial cells (Agnoletti et al., 1999; Valgimigli et al., 2003). Hence, this is a sub-analysis of The comparisoN between ticAgrelor and clopidogrel effect on endoTHelial platelet ANd iNflammation parameters in patiEnts with SCAD and COPD study, aiming to investigate the underlying mechanism of ticagrelor- mediated improvement of endothelial function, comparing the effect on cytokines level of 1 month treatment of ticagrelor vs. clopidogrel, and then assessing p-eNOS levels in endothelial cells treated with patient's sera and investigating possible correlations between cytokines changes and this marker of endothelial function.

## Materials and methods

### Study design/population/randomization and interventions

This is a sub-study of the clinical trial “The comparisoN between ticAgrelor and clopidogrel effect on endoTHelial platelet ANd iNflammation parameters in patiEnts with SCAD and COPD” undergoing PCI (NATHAN-NEVER). The clinical trial was registered at www.clinicaltrials.gov with the identifier NCT02519608. The protocol was approved by “Comitato Etico Unico della Provincia di Ferrara.” All subjects gave written informed consent in accordance with the Declaration of Helsinki. Details of the design of the study and the outcomes of the clinical trial are reported in Campo et al. (2017). Briefly, population inclusion criteria were: (1) age >18 years; (2) ability to provide informed written consent; (3) SCAD diagnosis with coronary artery angiography (CAA) and PCI; (4) COPD diagnosis confirmed by spirometry. Main exclusion criteria were: (1) prior administration of P2Y_12_ inhibitor (clopidogrel, ticlopidine, prasugrel, ticagrelor) or of anticoagulant drugs; (2) known intolerance to clopidogrel or ticagrelor; (3) prior intracranial hemorrhage; (4) cerebrovascular accident and/or active major bleeding and/or major surgery within the last 30 days and (5) other known inflammatory chronic disorders. Randomization /interventions: 46 patients were enrolled and randomly assigned to receive, on top of aspirin, clopidogrel, or ticagrelor. To minimize potential confounding effects, randomization was stratified according to the presence of diabetes and COPD severity.

### Inflammation parameters (cytokines/chemokines) analysis

Sera (*n* = 20 per group) were kept at −80°C and thawed only once before performing the MILLIPLEX MAP Human Cytokine/Chemokine Panel assay (Merck Millipore, Billerica, MA), a multiplex immunoassay, which allows the simultaneous detection and quantification of the following 29 human cytokines/chemokines: epidermal growth factor (EGF), Eotaxin, granulocyte colony-stimulating factor (G-CSF), granulocyte monocyte colony-stimulating factor (GM-CSF), interferon (IFN) -α2, IFN-γ, interleukin (IL)-10, IL-12(p40), IL-12(p70), IL-13, IL-15, 1L-17α, IL-1 receptor antagonist (ra), IL-1α, IL-1β, IL-2, IL-3, IL-4, IL-5, IL-6, IL-7, IL-8, inducible protein (IP)-10 (CXCL10), monocyte chemoattractant protein (MCP)-1, macrophage inflammatory protein (MIP)-1α, MIP-1β, tumor necrosis factor (TNF) -α, TNF-β, and vascular endothelial growth factor (VEGF). Samples were processed following the manufacturer's instructions and data were analyzed by MAGPIX instrument provided with the MILLIPLEX-Analyst Software.

### Platelet function analysis

On-treatment platelet reactivity (PR) was assessed by the VerifyNow™ system (Accumetrics, San Diego, CA, USA), using a specific assay to evaluate P2Y_12_ inhibitors (VerifyNow P2Y_12_™). The results were expressed as P2Y_12_ reaction unit (PRU).

### Cell culture

HUVECs pools, purchased from Life Technologies, were plated on 1.5% gelatin-coated tissue culture dishes and maintained in basal medium M200 (Life Technologies, Carlsbad, CA, USA) containing 2% FBS and growth factors (EGM-2, Life Technologies, Carlsbad, CA, USA) at 37°C with 5% CO_2_. Cells from passages 2 to 4 were actively proliferating (70–90% confluent) when samples were harvested and analyzed. EGM-2 contains EGF among other growth factors, in the experiments where HUVECs were treated with different concentrations of EGF cells were seeded and grown in EGM-2 without EGF and different concentrations of recombinant human EGF (rhEGF) were added 48 h prior to cell lysis.

### ELISA

Activation of eNOS in HUVEC was assessed by ELISA (Cell Signaling Technology, Danvers, USA) with antibody specific for the phosphorylated form of eNOS (p-eNOS Ser1177) following manufacturer instructions. Briefly, HUVECs were treated for 48 h with patient's sera (*n* = 18 per group) or were grown in M200 supplemented with EGM2 without EGF. Analysis of eNOS activation were performed after 48 h incubation with patient's sera or different concentrations of rhEGF. Protein concentration of each lysate was quantified by using Pierce BCA Protein Assay Kit (Thermo Scientific, Wilmington, USA) and 10 μg of total protein extract was used for each well. Data were expressed as p-eNOS levels as absorbance at 488 nm.

### Western blot

Western blot analysis was carried out to detect expression of total eNOS and β-actin as previously described (Fortini et al., 2017). Briefly, cells were lysed in RIPA buffer and protein concentration of each lysate was quantified by Pierce BCA Protein Assay Kit (Thermo Scientific, Wilmington, USA). The same amount of total protein (10 μg) was loaded in each lane, then proteins were separated on 7% NuPAGE gels (Life Technologies, Carlsbad, CA. USA). Proteins were transferred to PVDF membranes and were incubated overnight at 4°C with primary antibodies, washed three times in TBS/Tween 0.1%, and then incubated for 1 h at room temperature with secondary peroxidase-conjugated antibodies. Membranes were washed three times in TBS/Tween 0.1% and developed using Western Lightning ECL Pro (PerkinElmer, Waltham, MA). Images were obtained by exposing membranes to Chemidoc. Immunoreactive bands were analyzed with ImageLab analysis software (Bio Rad, Hercules, CA). Mouse antibody against eNOS was from BD Biosciences (Franklin Lakes, New Jersey, USA), mouse monoclonal against β-actin and secondary anti-mouse antibody were from Sigma-Aldrich (St. Louis, MS, USA). β-actin was used for normalization in the quantitative evaluation of western blots after verifying that β-actin levels were not affected by any treatment in comparison to total protein evaluated by staining with Ponceau (Sigma-Aldrich, Saint Louis, MO).

### Statistical analysis

Normal distribution of the variables was explored with the Kolmogorov–Smirnov test and with the Shapiro test (alpha = 0.05). Variables were presented with mean ± standard deviation or median ± interquartile range. Normally distributed variables were compared by *t*-test and one-way ANOVA; otherwise the Mann-Whitney U and Kruskal-Wallis tests were used. For comparisons between two groups two-tailed unpaired Student's *t*-tests were used except for the comparisons in the before and after analysis in which two-tailed paired Student's *t*-tests were used. When more than two groups were compared, one-way ANOVA with Student-Newman-Keuls method for multiple comparisons was used. Correlations between EGF and p-eNOS or, EGF and PR were assessed using Spearman correlation test (alpha = 0.05). In cells experiments with exogenous EGF results are expressed as mean ± *SD* of at least three independent experiments. Statistical analysis was perfomed with Graphpad Prism 6.0 (Graphpad Software, La Jolla, CA).

## Results

### Cytokines/chemokines

**Figure 1:**
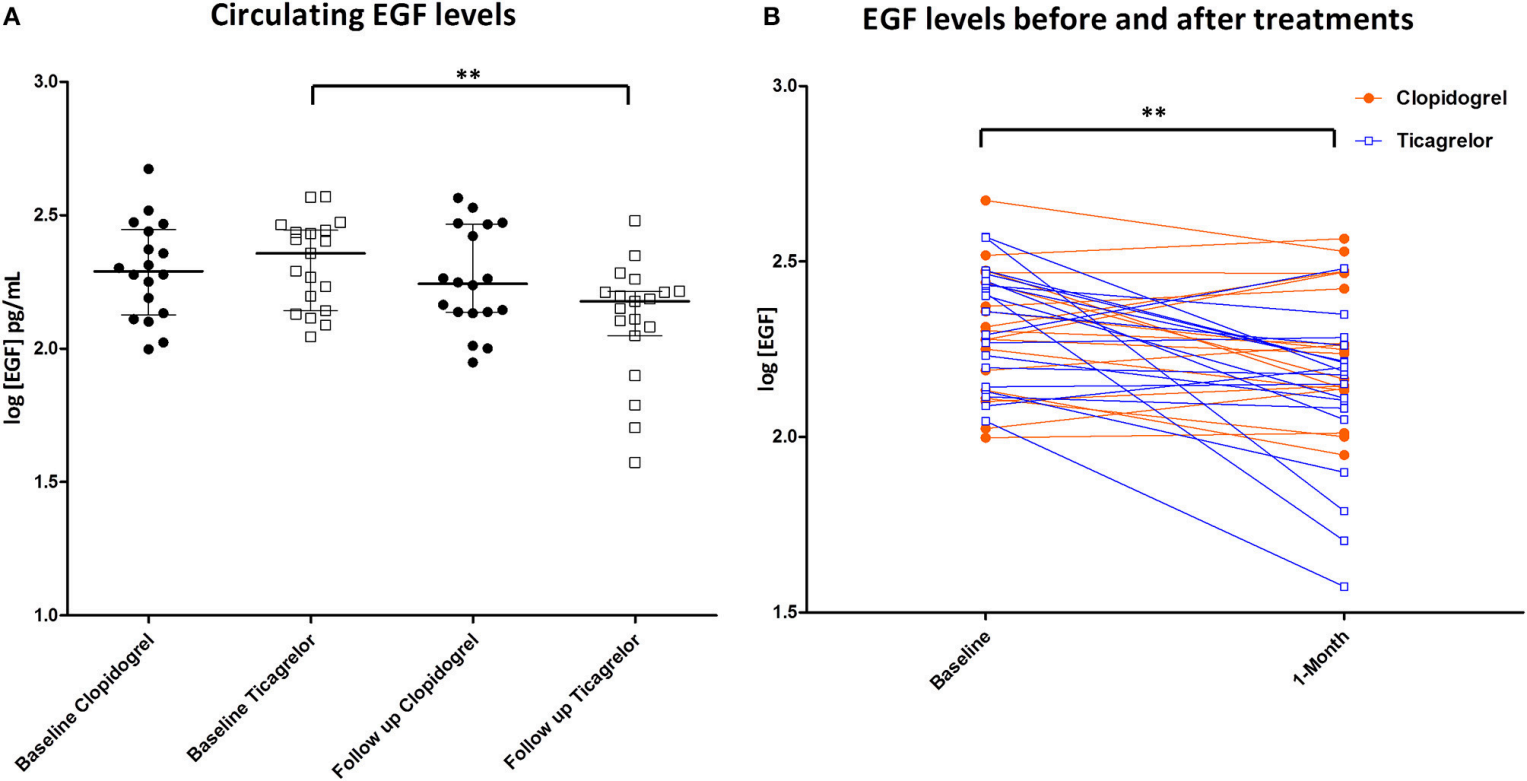
**(A)** EGF levels in the serum of patients at baseline and after 1 month of treatment. Black circle: patient randomized to clopidogrel. White square: patient randomized to ticagrelor. **P < 0.01; **(B)** EGF levels of patients representation before and after treatments. Orange circle: patient randomized to clopidogrel. Blue squares: patient randomized to ticagrelor. **P < 0.01.

As previously reported, after 1 month treatment of ticagrelor or clopidogrel, serum levels of inflammation-related cytokines/chemokines, in patients with concomitant SCAD and COPD undergoing PCI, did not statistically differ (Campo et al., 2017). In this study, the effect of each treatment on cytokine level was further investigated with a before and after statistical analysis. We found that there is a significant decrease in the serum levels of EGF in patients treated with ticagrelor, but not in the clopidogrel group. EGF serum concentration was log- normally distributed, as tested with the Kolmogorov–Smirnov test. Accordingly, EGF levels in the different groups were expressed as log[EGF] (Figures 1A,B) and further analyses were performed using log-transformed EGF values. Difference between log[EGF] means before and after treatment with ticagrelor was 0.21 (*p* < 0.01; 95% CI difference of means = 0.09–0.33).

### Correlation between EGF changes and platelet reactivity

**Figure 2:**
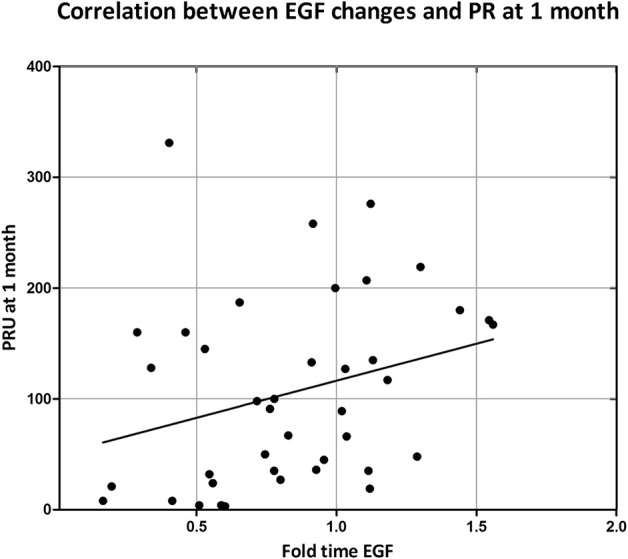
Correlation between on-treatment platelet reactivity at 1 month and EGF changes. On treatment platelet reactivity was measured by VerifyNow after and expressed as P2Y_12_ reaction unit (PRU). EGF changes were expressed as ratio between serum EGF concentration at 1 month over serum EGF concentration at baseline. Correlation was assessed by Spearman correlation test: *R* = 0.350; *p* = 0.020.

As previously reported, ticagrelor is more powerful in inhibiting PR compared with clopidogrel (Lemesle et al., 2015; Campo et al., 2017). After 1-month treatment, changes in EGF levels correlated with on-treatment platelet reactivity (*R* = 0.355; *p* = 0.020) (Figure 2).

### Changes in p-eNOS levels changes and correlation with EGF

**Figure 3:**
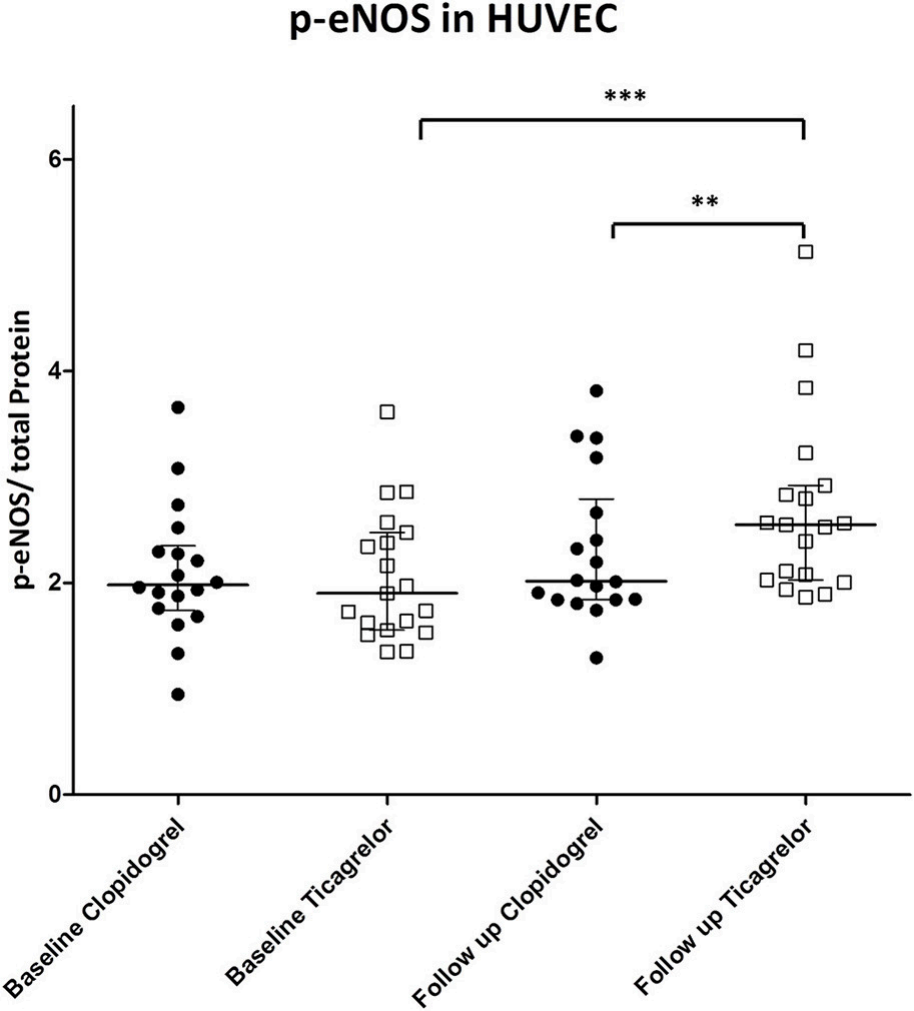
p-eNOS in HUVEC treated with patient's sera at baseline and after 1 month of treatment. Black circle: patient randomized to clopidogrel. p-eNOS levels are expressed as OD at 450 nm. White square: patient randomized to ticagrelor. **P < 0.01; ***P < 0.001.

In HUVECs treated with patient's sera we found an increase of p-eNOS only in those of patients treated with ticagrelor at 1 month compared to the baseline (OD 2.0 at baseline vs. 2.7 after 1 month; *p* < 0.01). Furthermore, we observed a significant difference in p-eNOS levels between HUVEC treated with the sera of the two treatment groups (OD 2.1 vs. 2.7, clopidogrel, and ticagrelor, respectively; *p* < 0.01) (Figure 3).

**Figure 4:**
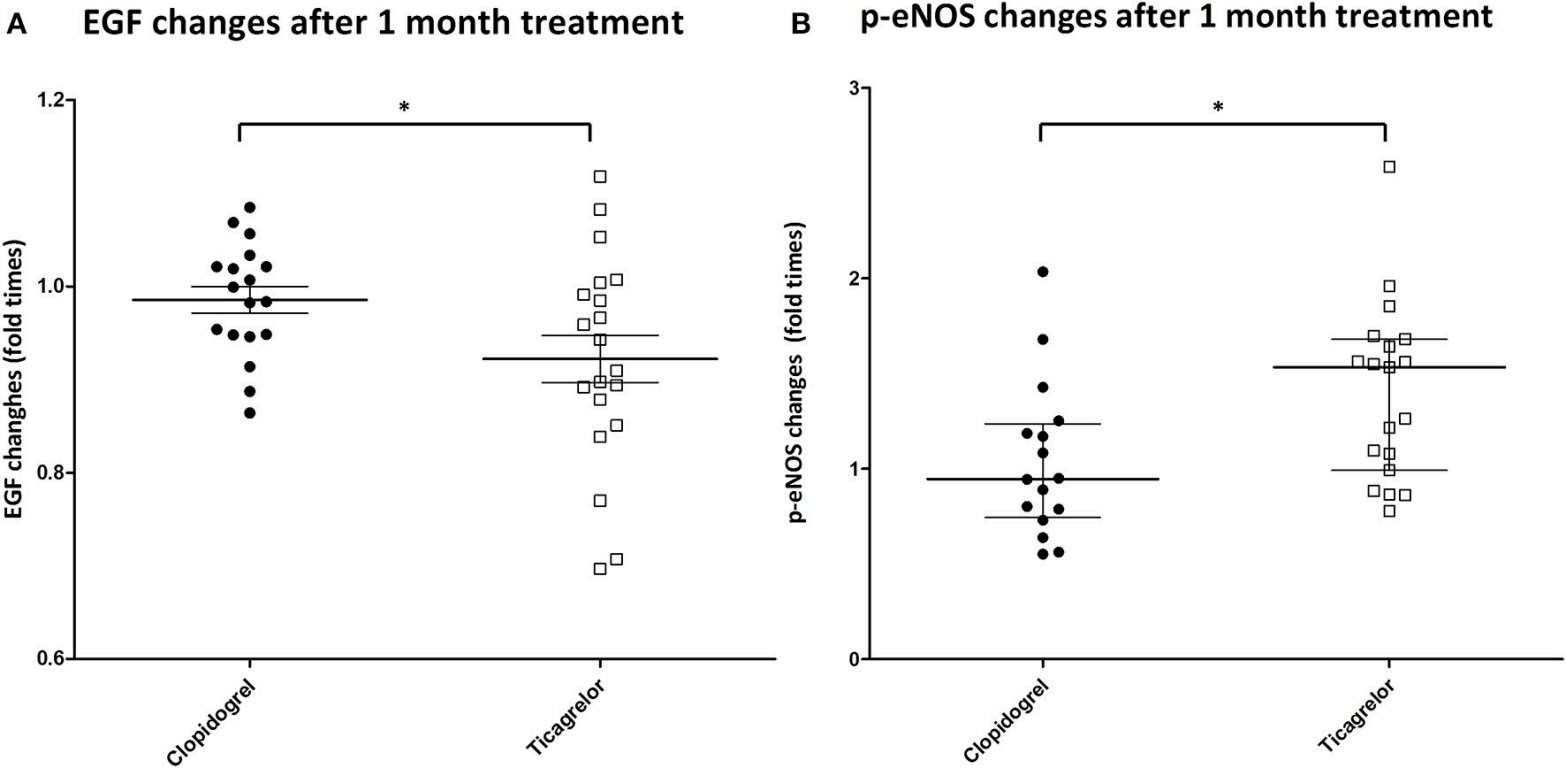
**(A)** Changes in EGF levels in the serum of patients (on the y-axis is shown the ratio between EGF levels after 1 month of treatment and at baseline). Black circle: patient randomized to clopidogrel. White square: patient randomized to ticagrelor. *P < 0.05. **(B)** Changes in p-eNOS levels in HUVEC treated with patient's sera (on the y-axis is shown the ratio between p-eNOS levels in HUVEC treated with patient's sera after 1 month of treatment and at baseline). Black circle: patient randomized to clopidogrel. White square: patient randomized to ticagrelor. *P < 0.05.

**Figure 5:**
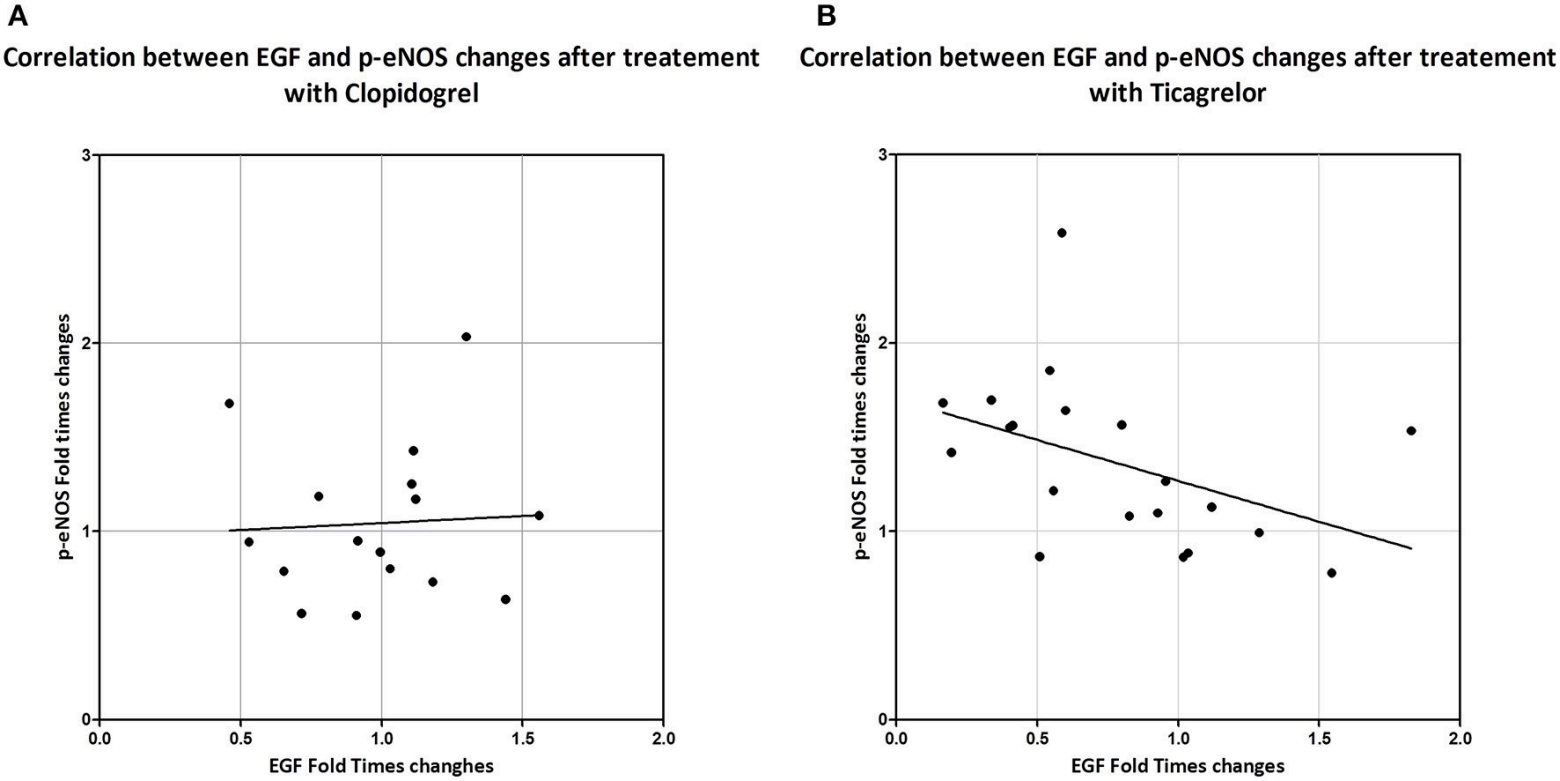
Correlation between changes in levels of circulating EGF (ratio between EGF levels after 1 month of treatment and at baseline) and of p-eNOS in serum treated HUVEC (ratio between p-eNOS levels in HUVEC treated with patient's sera after 1 month of treatment and at baseline) in patients treated with clopidogrel **(A)** or with ticagrelor **(B)**. Correlation was assessed by Spearman correlation test. Clopidogrel: *R* = 0.100; *p* = 0.714. Ticagrelor: *R* = −0.571; *p* = 0.002.

We then analyzed the changes in EGF and p-eNOS levels after 1 month treatment with the two different antiplatelet drugs. The efficacy in modulating EGF or p-eNOS of clopidogrel or ticagrelor was expressed as ratio of EGF or p-eNOS levels after 1 month over the respective EGF or p-eNOS at baseline. Fold-times changes of EGF (EGF_1month_/EGF_baseline_) and of p-eNOS (p-eNOS_1month_/p-eNOS_baseline_) are shown in Figures 4A,B. Ticagrelor was shown to be more powerful in decreasing EGF compared to clopidogrel (*p* = 0.042) and also more efficient in increasing p-eNOS (*p* = 0.020). In addition, correlation analyses between changes in EGF and in p-eNOS levels (Figures 5A,B) showed a correlation between EGF and p-eNOS ratio in the ticagrelor (*R* = −0.571; *p* = 0.002) but not in the clopidogrel group (*R* = 0.100; *p* = 0.714).

### Effect of exogenous EGF on eNOS in HUVECs

**Figure 6:**
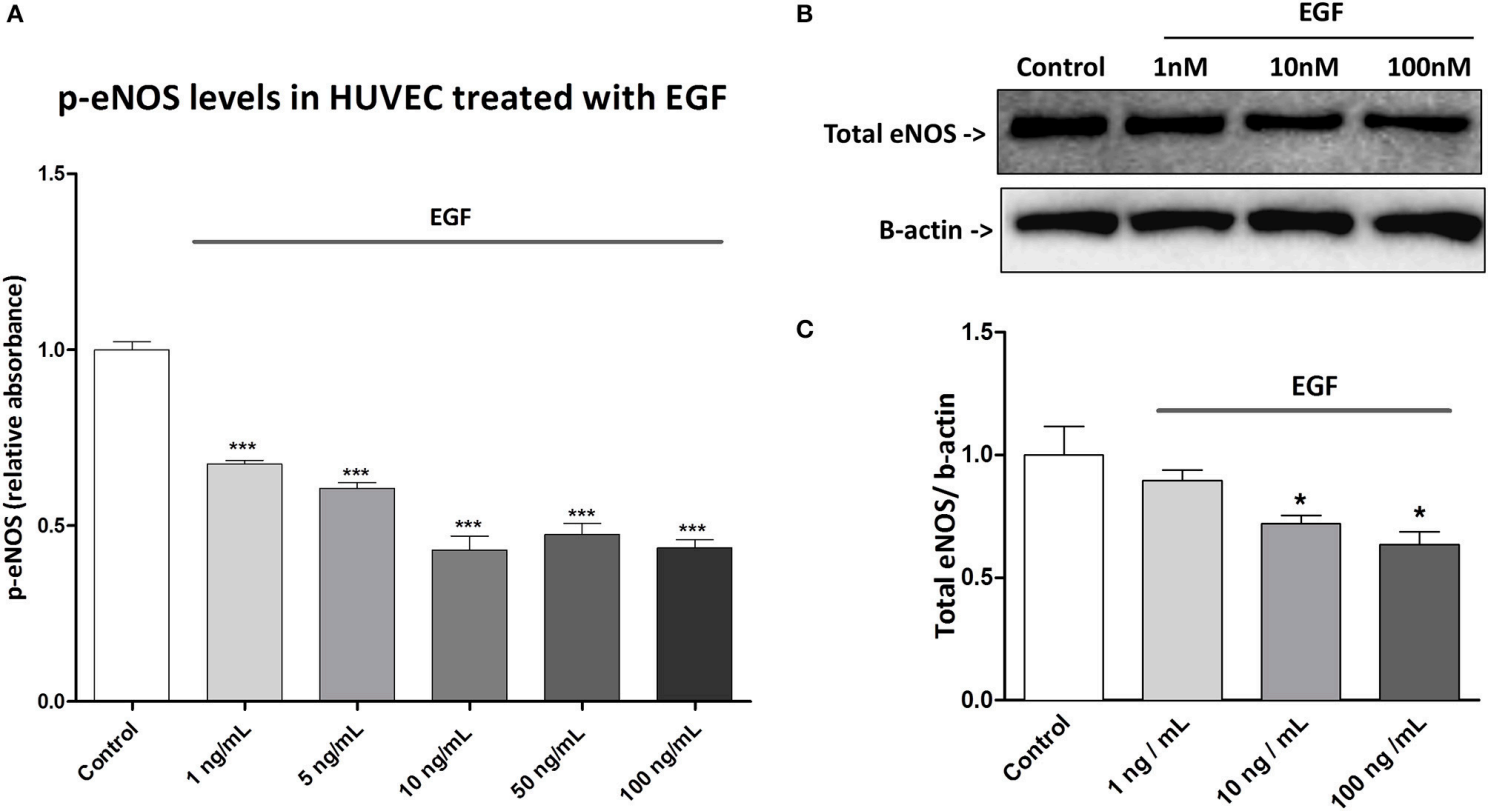
Effect of exogenous EGF in HUVEC. **(A)** HUVECs were treated for 48 h with different concentrations of EGF ant then were lysed. Proteins were quantified by BCA assay and 10 μg of protein were used for each well in the ELISA. p-eNOS levels were expressed as absorbance (OD) at 450 nm. Results are expressed as mean ± SD of three independent experiments. ***P < 0.001 (comparison between control and treatment). **(B)** HUVECs were treated for 48 h with EGF. Lysates were electrophoresed and immunoblotted with total eNOS antibody, β-actin antibody was used to ensure equal loading. Images are representative of one of three independent experiments. **(C)** Densitometric analysis of western blot. *P < 0.05 (comparison between control and treatments).

To assess a possible causal relationship between EGF levels and eNOS activation we treated HUVEC with different concentrations of EGF and we evaluated p-eNOS levels. We found that p-eNOS levels quantified by ELISA were decreased by EGF in a dose-dependent manner (Figure 6A). In addition, we analyzed by western blotting the total eNOS content in HUVEC treated with different concentration of EGF and we found that also total eNOS protein was diminished by EGF in a dose-dependent manner (Figure 6B).

## Discussion

In this study we report that, in patients with SCAD and COPD undergoing PCI, 1 month treatment with ticagrelor, but not with clopidogrel, results in a decrease of serum EGF concentration (Figures 1A,B). EGF can be secreted in the blood stream by different cellular sources such as submandibular gland (Tsutsumi et al., 1986), kidney (Staruschenko et al., 2013), and platelets (Ben-Ezra et al., 1990; Feng et al., 2012). However, the individual contribution of the different sources of circulating EGF as well as how the EGF levels are physiologically determined are not known.

EGF is a small protein constituted by 53 amino acids and three intramolecular disulfide bridges, and it is the founding member of the EGF-family of proteins. EGF acts by specifically binding to epidermal growth factor receptor (EGFR) on the cell membrane (Carpenter and Cohen, 1990). EGFR is a transmembrane receptor that controls signal transduction pathways that ultimately lead to survival, differentiation, and/or cell proliferation (Zeng and Harris, 2014). Activation of EGFR is known to be involved in many pathological processes such as endothelial dysfunction (Mehta et al., 2008), hypertension (Zhou et al., 2009), restenosis (Chan et al., 2003; Shafi et al., 2009), atherogenesis (Dreux et al., 2006), and cardiac remodeling (Iwamoto et al., 2003). More recently, it has been also shown that EGFR inhibition in T cells reduces atherosclerosis development (Zeboudj et al., 2018). The precise contribution of specific EGFR ligands is still poorly understood in the cardiovascular context but different lines of evidence have revealed that EGF can negatively affect endothelial function (Belmadani et al., 2008; Kassan et al., 2015), vascular tone regulation (Lundstam et al., 2007; Sumaya and Storey, 2017) and that it provokes proliferation of vascular smooth muscle cells (Berk et al., 1985).

We found a correlation, even if not particularly strong (*R* = 0.350), between changes in serum EGF concentrations and on-treatment platelet reactivity after pharmacological treatment (Figure 2). Certainly, other factors could be involved in the level of PR and this will be the object of our future studies. EGF is present in platelets α-granules from where it can be secreted in the blood stream following degranulation (Lev-Ran et al., 1990; Blair and Flaumenhaft, 2009; Bertrand-Duchesne et al., 2010; Durante et al., 2013). Notably, it is known that inhibiting P2Y_12_ receptors not only blocks platelet aggregation but also reduces release of mediators from α-granules (Zhao et al., 2001; Storey et al., 2002). In this study, we did not investigate the mechanism by which EGF is decreased by ticagrelor, but based on the current knowledge, it appears likely that the observed decrease in the serum EGF could be due to a decrease in the EGF released by platelets.

Moreover, we showed that in HUVEC treated with sera of patients of the ticagrelor group at 1 month there is a higher activation of eNOS, in comparison to clopidogrel at the same time point (Figure 3). Interestingly, we found a negative correlation between changes in levels of circulating EGF and in eNOS activation in the ticagrelor arm (Figure 5B), but not in the clopidogrel group (Figure 5A). Taken together our data suggest that EGF interferes with eNOS activation. The role of EGF in decreasing eNOS activity has already been reported in animal models: in diabetic mice EGFR activation, resulting in eNOS activity impairment, is implicated in mesenteric resistance artery (MRA) dysfunction (Belmadani et al., 2008); in the same model, endogenous EGF decreased p-eNOS levels (Kassan et al., 2015). Here we showed *in vitro* that EGF is able to decrease both eNOS phosphorylation (Figure 6A) and total eNOS protein (Figures 6B,C) in a dose-dependent manner in HUVECs. Taken together our data indicate that ticagrelor is able to reduce circulating EGF levels and that, in turn, lower serum EGF positively affects the endothelial function by facilitating eNOS activity.

In conclusion, we showed that the positive effect of ticagrelor on endothelial function is, at least in part, mediated by its capacity to lower EGF that in turn results in a better eNOS activation. Our data suggest that lower platelet reactivity could result in lower EGF release by platelets through a P2Y_12_-mediated mechanism. This would imply that ticagrelor improvement of endothelial function is not only due to mechanisms linked to increased adenosine availability but could also be directly connected to its higher efficacy in diminishing platelet reactivity.

## Ethics statement

The protocol has been approved by the corresponding Ethics Authority (Comitato Etico Unico della Provincia di Ferrara). All patients gave their written informed consent. The study is registered at www.clinicaltrials.gov with the identifier NCT02519608.

## Author contributions

FVDS and FF: Performed *in vitro* experiments; FVDS and PR: Analyzed the data and wrote the paper; GC and RF: Designed and supervised the clinical trial and provided critical revision of data; GA, RP, SB, DB, and ADF: Participated to clinical assessments, collection of samples, and laboratory analyses. All authors reviewed data and results, and approved the final version of the manuscript.

### Conflict of interest statement

GC has received honoraria for lectures from Astrazeneca, Menarini, Abbott Vascular, Boston Scientific. RF has received honoraria for lectures from Servier. The other authors declare that the research was conducted in the absence of any commercial or financial relationships that could be construed as a potential conflict of interest.
